# Collagen/Chitosan Gels Cross-Linked with Genipin for Wound Healing in Mice with Induced Diabetes

**DOI:** 10.3390/ma15010015

**Published:** 2021-12-22

**Authors:** Balzhima Shagdarova, Mariya Konovalova, Yuliya Zhuikova, Alexey Lunkov, Vsevolod Zhuikov, Dolgor Khaydapova, Alla Il’ina, Elena Svirshchevskaya, Valery Varlamov

**Affiliations:** 1 Research Center of Biotechnology, Russian Academy of Sciences, 119071 Moscow, Russia; shagdarova.bal@gmail.com (B.S.); zhuikova.uv@gmail.com (Y.Z.); fwnf1994@gmail.com (A.L.); vsevolod1905@yandex.ru (V.Z.); ilyina@biengi.ac.ru (A.I.); 2 Shemyakin-Ovchinnikov Institute of Bioorganic Chemistry, Russian Academy of Sciences, 117997 Moscow, Russia; mariya.v.konovalova@gmail.com (M.K.); esvir@ibch.ru (E.S.); 3 Faculty of Soil Science, M.V. Lomonosov Moscow State University, 119234 Moscow, Russia; dkhaydapova@yandex.ru

**Keywords:** chitosan, collagen, gels, genipin, wound healing

## Abstract

Diabetes mellitus continues to be one of the most common diseases often associated with diabetic ulcers. Chitosan is an attractive biopolymer for wound healing due to its biodegradability, biocompatibility, mucoadhesiveness, low toxicity, and hemostatic effect. A panel of hydrogels based on chitosan, collagen, and silver nanoparticels were produced to treat diabetic wounds. The antibacterial activity, cytotoxicity, swelling, rheological properties, and longitudinal sections of hydrogels were studied. The ability of the gels for wound healing was studied in CD1 mice with alloxan-induced diabetes. Application of the gels resulted in an increase in VEGF, TGF-b1, IL-1b, and TIMP1 gene expression and earlier wound closure in a comparison with control untreated wounds. All gels increased collagen deposition, hair follicle repair, and sebaceous glands formation. The results of these tests show that the obtained hydrogels have good mechanical properties and biological activity and have potential applications in the field of wound healing. However, clinical studies are required to compare the efficacy of the gels as animal models do not reproduce full diabetes pathology.

## 1. Introduction

Diabetes mellitus continues to be one of the most common diseases in the world, despite the fact that it has been studied and treated for many years. The prevalence of the disease among adults in 2017 was 8.8% of the global population, which is 425 million people [1]. In diabetes mellitus, the development of peripheral vascular disease due to poor blood circulation is often observed. Diabetic neuropathy of the skin is associated with the destruction of nerve endings, which leads to excessive skin vulnerability. Minor injuries (bruises, cracks, calluses, abrasions) also lead to serious problems. The progression of the disease results in the formation of chronic, long-term non-healing ulcers, purulent wounds, and in the case of untimely treatment, to a risk of foot amputation [2,3].

It is known that various materials are used to treat wounds, including biopolymers [4,5,6]. Among them, chitosan is an attractive polymer due to a variety of natural sources of production, biodegradability, biocompatibility, mucoadhesiveness, low toxicity, hemostatic effect, and a number of other biological activities [7,8]. Structurally, chitosan is a heteropolysaccharide consisting of alternating glucosamine and N-acetylglucosamine units connected by β-(1-4) glycosidic bonds, where glucosamine is a dominant link in the macromolecule chain. Biocompatibility with damaged tissues, as well as its ability to “open” tight junctions, is a necessary property for the development of topical forms [9,10]. Chitosan, unlike most polysaccharides, has a positive charge at the physiological pH of the skin, which allows it to interact with the negatively charged surface of skin cells. By binding to epidermal cells, chitosan can form water-retaining coatings that help to maintain optimal moisture content in the affected areas, which contributes to the healing [11]. On the basis of chitosan and its derivatives, nonwovens, sponges, sprays, gels, hydrogels, and membranes are produced. Such materials are very promising for wound healing [12].

Collagen is a fibrillar protein that forms the basis of the body’s connective tissue and ensures its strength and elasticity. Collagen performs a morphogenetic function, influencing the growth, migration, differentiation, secretory, and synthetic activity of various cells. When used in wound healing materials, the formation of collagen fibers by the body itself is initiated, which accelerates the regeneration of tissue and skin cells. Typically, for the production of neoplastic agents, native non-reconstructed type I collagen isolated from the treated dermis of cattle is used [13,14,15]. The main disadvantages of collagen used as wound healing material are poor mechanical properties and rapid degradation, for example when used in tissue engineering [13]. However, the use of crosslinking agents, or the formation of gels with polysaccharides, is effective in solving these problems.

Various crosslinking agents are used to crosslink gels: Glutaricdialdehyde, EDC-NHS, riboflavin, transglutaminase, dehydrothermal treatment, UV, and genipin. Genipin has been used predominantly due to a number of benefits. It is a plant-based agent that has a good level of crosslinking, and increases the modulus of elasticity [16,17]. Genipin exhibits antioxidant, anti-inflammatory, antitumor, antifungal, antidiabetic activity, and inhibits the proliferation of certain cancer cells, including leukemia, breast cancer, prostate cancer, and hepatocellular cancer [18]. Genipin is a well-studied substance in the field of biomedical applications [13,18,19,20]. The safety and beneficial properties of genipin have been proven by a number of research projects in the treatment of diabetes, periodontitis, cataracts, liver dysfunction, as well as in wound healing and nerve tissue regeneration [21].

It is known that an approach based on the addition of metal nanoparticles to the structure of polymer gels can be used to accelerate the healing of wounds [22,23]. Metal nanoparticles have high specific surface area and a size of 1–100 nm. Silver nanoparticles (AgNPs) are characterized by a wide spectrum of antimicrobial activity and are the most studied [24,25]. However, despite the unique properties of metal nanoparticles, the currently used methods of their synthesis do not provide a sufficient reduction in toxicity, which limits their use in biomedicine. The formation of AgNPs using biopolymers leads to a decrease in their toxicity and an improvement in biocompatibility with living tissues [26]. Various polysaccharides are used in the synthesis of nanoparticles as surface modifiers and stabilizers [27]. AgNPs have been shown to promote faster wound closure. This is accomplished by stimulating the proliferation and migration of keratinocytes and controlling the differentiation of fibroblasts into myofibroblasts. Liu et al. reported that AgNPs have bactericidal, anti-inflammatory effects and promote wound healing, potentially regulating fibroblast migration and macrophage activation [28].

Thus, the main requirements for new materials used for wound healing in patients with diabetes mellitus are biocompatibility, low cytotoxicity, and the presence of antimicrobial action [29,30]. In this context, this study aimed at obtaining hydrogels based on chitosan, collagen, and silver nanoparticles, studying their antibacterial activity, cytotoxicity, swelling, rheological properties, and determining their wound healing diabetic wounds.

## 2. Materials and Methods

### 2.1. Materials

Chitosan with molecular weight (MW) 1040 kDa and degree deacetylation (DD) 85% (Bioprogress LLC, Schelkovo, Moscow region, Russia), collagen type I 1% solution (Sheneskin, Ulan-Ude, Russia), genipin (Sigma, St. Louis, MO, USA), acetic acid, alloxan, phosphate buffered saline, and all other reagent were of the highest purity grade commercially available (Sigma, St. Louis, MO, USA).

### 2.2. Preparation of Chitosan

Chitosan for the formation of gels was obtained by the method [31]. High-molecular chitosan was suspended in dilute nitric acid. The resulting suspension was heated to 70 °C during stirring, hydrolysis was carried out for 8 h, then the mixture was kept without stirring for 16 h at 23 °C. The precipitate obtained after hydrolysis was separated by filtration on a porous glass filter, and then washed with isopropyl alcohol to remove nitric acid residues. Next, the product was dialyzed using dialysis membranes (MWCO: 12.000–14.000) with multiple changes of distilled water. Then the product was freeze-dried in a freeze dryer Martin Christ ALPHA 1-4LD plus (Christ, Osterode am Harz, Germany). Depending on the concentration of nitric acid, chitosan with a molecular weight (28–700 kDa) was obtained.

### 2.3. Characterization of Chitosan

The physico-chemical characteristics of chitosan were determined. The average molecular weight and polydispersity index of chitosan were determined by high-performance gel-penetrating chromatography (HPLC) on the S 2100 Sykam chromatograph (Sykam GmbH, Eresing, Germany) on a column (7.8 mm × 300 mm) Ultahydrogel-250 (Waters Corporation, Milford, MA, USA) with a pre-column (4 × 3 mm) GFC-4000 (Phenomenex, Torrance, CA, USA).

^1^H NMR spectra of chitosans were recorded on a Bruker AMX 400 spectrometer (Bruker, Billerica, MA, USA) operating at 1H frequency of 400 MHz at 32 °C. Samples were prepared in deuterium water. 4,4-Dimethyl-4-silapentane-sulfonic acid was used as a standard. The solvent signal was suppressed by selective pulses using gradients.

#### Antibacterial Tests

To determine the minimum inhibitory concentration (MIC) of chitosan solutions *Escherichia coli* ATCC 25922, *Staphylococcus aureus* ATCC 35591 and *Staphylococcus epidermidis*33 GISK were used. Bacterial cultures were stored in LB-agar (LB Agar Miller) at 4 °C. To prepare the inoculum, a single colony of bacteria was transferred into 20 mL of LB (LB Broth Miller) and incubated on a shaker at 150 rpm for 18 h at 37 °C. The bacterial suspension in the nutrient medium was added to serial two-fold dilutions of chitosan, and the microbial load was 10^5^ cells/mL. MIC was defined as the lowest concentration required to suppress cell proliferation [32].

To determine the antifungal activity, the *Candida albicans* 90028 strain was used. The isolated yeast was grown and stored on Sabouraud dextrose agar at 4 °C. To prepare the inoculum, a single yeast cell colony was transferred into 20-mL Sabouraud broth and shaken for 18 h at 150 rpm. A cell suspension was added to serial two-fold dilutions of chitosan; the load was 10^3^ cells/mL. MIC was defined as the lowest concentration required to suppress cell proliferation [33]. 

### 2.4. Preparation of Silver Nanoparticles 

Silver nanoparticles were obtained using quaternized chitosan with gallic acid (NPs) as a stabilizing and reducing agent as described in [34]. Quaternized chitosan was obtained using glycidyltrimethylammonium chloride according to the method [35], then using carbodiimide and N-hydroxysuccinimide, gallic acid was cross linked to the chitosan derivative, obtaining quaternized chitosan with gallic acid (QCG) according to the method [36]. Next, 10 mg of QCG was dissolved in 10 mL of distilled water, the solution was titrated to pH = 7.5 with an aqueous solution of ammonia, then 2 mL of AgNO_3_ solution (2 mg/mL) was added to the resulting solution. The resulting reaction mass was dialyzed against water in a MWCO 12,000–14,000 dialysis tube (Spectrum Laboratories Inc., Compton, CA, USA) for 24 h.

### 2.5. Preparation of Gels

Gels were obtained using a modified method from [37]. We used a 1% (*w/v*) chitosan solution with MM 100 and 700 kDa in a 1% acetic acid solution and a 1% (*w/v*) collagen solution. Gels were obtained by mixing these solutions in different ratios: 1:0, 1:1, 1:3, 1:5, 0:1 (chitosan:collagen). Then the samples were frozen and lyophilized in a freeze dryer. Thereafter, the dried gels were washed in ethanol for 1 h. To crosslink the gels, we used a solution of genipin (g) of various concentrations (3, 0.3, and 0.03%) in a phosphate buffer saline solution (PBS, pH 7.4). Genipin solution (3 mL) was added to each gel and left for 24 h at room temperature. The next day, the genipin-cross-linked gels were washed with distilled water and 200 μL of NPs suspension was added. Then the gels with NPs were frozen and lyophilized in a freeze dryer.

### 2.6. Swelling Test

Swelling capacity was assessed by gravimetric analysis. Freeze-dried gel samples were preliminarily weighed (*w*_0_), then immersed in Petri dishes with distilled water or a simulated biological fluid—PBS, pH 7.4. After 1 h and 24 h, it was separated from the liquid medium in a swollen state, excess moisture was removed with filter paper, and the mass of the swollen gel (*w*) was determined. The swelling capacity [g/g] was determined on the basis of Equation (1):

(1) $$Swelling\;ratio=\left(w-w_{0}\right)/w_{0}$$

where: *w* is the mass of the swollen sample [g], and *w*_0_ is the mass of the hydrogel before immersion in the solution [g] [38].

### 2.7. IR Spectroscopy and SEM Characterization

IR spectra were recorded on a Perklin-Elmer 1420 spectrometer (Perklin-Elmer, Hopkinton, MA, USA) covering the frequency range from 4000 to 400 cm^−1^. The studied samples were prepared in KBr. 

The samples were frozen and lyophilized in a freeze dryer. The freeze-dried hydrogels were cut vertically and images of uncoated specimens were taken on a Tescan Vega 3 scanning electron microscope (SEM) (Tescan, Brno, Czech Republic) operated at low vacuum, at 10 kV (accelerating voltage), using a backscattered electron (BSE) detector. 

### 2.8. Rheological Measurements

The experimental evaluation of the viscoelastic properties of hydrogels was carried out by the oscillatory rheometry in shear mode using an MCR-302 rheometer (Anton Paar, Graz, Austria). All measurements were made on wet samples at 20 °C. We used a “plate-plate” measuring geometry (with a diameter of 25 mm), and the swollen sample diameter was always 25 mm, while sample thickness varied from 1.0 to 3.0 mm. The gap size between the rheometer’s lower and upper plates during the measurement was selected in accordance with the work [39].

Previously, an amplitude test was performed. An amplitude test is required to determine the area of linear viscoelasticity. This is important because during dynamic tests in this area, there is no mechanical destruction of the samples and their internal structure is preserved. The sample was subjected to oscillating voltages with a certain frequency (Angular Frequency = 10 rad/s) and varying amplitude. The value of the deformation amplitude at which the measurements did not go beyond the linear viscoelastic region was taken as 1% for all samples. To study the viscoelastic properties, a frequency sweep was used in the range of 0.1–100 rad/s with a fixed amplitude.

### 2.9. Cytotoxicity of Gels

To assess the cytotoxicity of the gels, MTT analysis was performed on human umbilical vein endothelial cells Ea.hy926. These cells were seeded in 96-well plates at 4 × 10^3^ per well, 100 μL of DMEM (containing 7% FBS) was added and incubated at 37 °C under 5% CO_2_. The gels were pre-sterilized by UV irradiation for 2 h and kept in 100 μL of a DMEM solution for 12 h. Gels 5 mm × 5 mm in size were placed in wells with immobilized endothelial cells a day later. The gels were incubated with the cells for 3 days. The metabolic activity of cells was assessed according to [40]. After sampling the gels, 100 μL of DMEM and 10 μL of MTT (5 mg/mL in PBS) were added to the wells with cells and incubated at 37 °C for 3 h. Then, the medium was removed from the plate, after which the formazan crystals were dissolved in 100 μL of DMSO (Reakhim, Moscow, Russia) for 20 min. Then the optical density was measured at 540 nm on a plate spectrophotometer Multiskan FC (Termo Fisher Scientific, Waltham, MA, USA). The data were presented in the form of an inhibition index calculated according to Equation (2):

(2) $$Inhibition\;index=1-OD_{exp}/OD_{cont},$$

where: *OD_exp_* and *OD_cont_* means optical density (*OD*) of experiment and control accordingly.

### 2.10. In Vivo Healing Assays

#### 2.10.1. Modeling Diabetes in Mice Using Alloxan

The study was carried out in male CD1 mice. CD1 mice (40 ± 5 g) were obtained from the Pushchino laboratory animal nursery, in the Moscow region. All animals had free access to food and water and were kept under conventional conditions. The protocol of the study was approved by the Ethics Committee of the Institute of Bioorganic Chemistry, the Russian Academy of Sciences, protocols #232 (2018) and #327 (2021). Before the administration of alloxan, the mice were starved overnight. Diabetes was induced by intravenous injection of alloxan (65 mg/kg body weight) freshly dissolved in sterile saline. Diabetes development was controlled through the blood glucose level 72 h after administration of alloxan using an Accu-Chek glucometer. For the study, we selected the mice with a blood glucose level >300 mg/dL (16.8 mM/L) [41].

#### 2.10.2. Modeling Wound Healing in Mice

Mice were operated under telazol/xylazine anesthesia. The back hair was removed with depilatory cream. After hair removal, the skin was treated with iodine and alcohol. On the dorsum, on either side of the midline, two round pieces of skin, 5 mm in diameter, including panniculosus carnosus, were carefully removed to create full thickness wounds. Silicone splints were applied and fixed with glue along the perimeter of the wounds. Then the splints were fixed with sutures. The wound healing material was applied to the wound on the left side of the back of the mouse. The wound on the right side served as a control without healing gels [42]. The wound area was measured with a digital vernier caliper (Enkor, Voronezh, Russia). Measurements were taken daily.

The percentage of wound healing was calculated using Equation (3):

(3) $$Wound\;healing=\frac{\left(Initial\;wound\;area\right)-\left(Wound\;area\;at\;specific\;time\;point\right)}{Initial\;wound\;area\;}*100\%.$$

### 2.11. Analysis of Gene Expression

#### 2.11.1. Gene Expression

Tissue samples taken near the wound collected at different days homogenized in 1 mL of ExtractRNA solution according to the manufacturer’s instruction (EuroGene, Moscow, Russia). Genomic DNA was removed by RNA-free DNAse digestion according to the manufacturer’s instruction (ThermoScientific, Waltham, MA, USA).

Synthesis of cDNA from the isolated mRNA was carried out using a commercial kit of reagents “Reverse transcriptase M-MuLV—RH” (BIOLABMIX LLC, Novosibirsk, Russia) containing the enzyme MuLVrevertase, deoxynucleotide triphosphates, a buffer for the enzyme, using a random hexaprimer from the same kit according to the manufacturer’s protocol. The resulting cDNA was stored at −20 °C until PCR was performed.

#### 2.11.2. Analysis of Gene Expression by Real-Time Polymerase Chain Reaction (qPCR)

A commercial kit with a ready-made PCR mixture BioMaster HS-qPCR SYBR Blue (2×) (BIOLABMIX LLC, Novosibirsk, Russia) was used to set up the polymerase chain reaction in real time according to the manufacturer’s protocol. The reaction was performed in a volume of 20 μL using specific primers. A list of primers is shown in Table 1.

The expression of MMP-1 and MMP-9 was also studied however no differences were found (data not shown). To carry out the reaction, we used an amplifier with real-time detection CFX Connect (Bio-Rad, Hercules, CA, USA) according to the following protocol: Initial denaturation for 5 min at 95 °C and then 40 cycles: 10 s—denaturation at 95 °C, 10 s—primer annealing at 60 °C, and 10 s—elongation at 72 °C. The qPCR results were processed using the CFX Manager software (Bio-Rad, Hercules, CA, USA). Analysis of the expression of each gene was performed in triplicate. The qPCR data were normalized to a GAPDH gene expression.

### 2.12. Histological Analysis of Wound Tissue 

Tissue samples were fixed with 4% paraformaldehyde and embedded in paraffin. Samples were cut into sections with a thickness of 4 μm using Leica RM 2145 RTS microtome (Leica Biosystems, Wetzlar, Germany). After dewaxing the tissue sections, they were stained with a commercial kit “Masson’s staining with aniline blue” (OOO Biovitrum, Saint Petersburg, Russia) according to the manufacturer’s protocol. Then the sections were dehydrated, clarified, and enclosed under a cover glass using the Consul-Mount histological medium. The stained samples were examined using light microscopy Zeiss Primo Star (Carl Zeiss, Oberkochen, Germany).

#### Statistical Analysis

The results were assessed using the methods of variation statistics using the standard statistical programs, Microsoft Excel and Statistica for Windows Version 6.0 (Stat Soft Inc., Austin, TX, USA). The mean, standard deviation (SD), standard error of the mean (SEM), and probability value (*p*) were determined. To assess the significance of differences between the samples, the Student’s *t*-test and the Mann Whitney U-test was used. Differences between the two compared values were considered statistically significant if the probability of their identity was less than 5% (*p* < 0.05).

## 3. Results and Discussion

### 3.1. Chitosan Properties

Chemical depolymerization of high molecular weight chitosan with a molecular weight (MW) of 1040 kDa and a degree of deacetylation (DD) of 85% was carried out using nitric acid. Depending on the concentration of nitric acid, chitosans with different molecular weights of 28, 54, 100, and 700kDa were obtained. The DD of the samples during hydrolysis increased to 93% (Table 2).

It is known that chitosans with a molecular mass of more than 100 kDa are most suitable for the production of films, various types of gels, and other applications [43]. To obtain hydrogels, we used samples with MW of 100 and 700 kDa.

The antibacterial and antifungal activities of the selected chitosans demonstrated the highest activity against *C. albicans*. The MIC for *E. coli* and *S. aureus* averaged ≥500 μg/mL (Table 3).

### 3.2. Preparation of NPs

It is known that metal nanoparticles enhance antibacterial activity, largely due to their size, which allows them to fully interact with the surface of bacterial cells. However, the main disadvantage of such nanoparticles is cytotoxicity [44]. To improve the biocompatibility of AgNPs, in our earlier study we synthesized AgNPs using quaternized chitosan with gallic acid [34]. The synthesized NPs were positively charged, the ζ-potential of the particles was 29 mV, their size was 13 and 45.9 nm as estimated by TEM and DLS, respectively (see Supplementary Materials). The resulting NPs not only had antibacterial activity against *E. coli* (250 μg/mL) and *S. epidermidis* (7.8 μg/mL), but also had low toxicity against epithelial cells. The inclusion of NPs in the nanocomposite based on the chitosan derivative reduced the cytotoxic effect of silver nanoparticles and retained their antibacterial activity.

### 3.3. Preparation of Gels

Gels were obtained from collagen and chitosan 100 and 700 kDa with various ratios of biopolymers (Table 4). Genipin was used as a cross-linking agent due to its lower toxicity compared to glutaricdialdehyde, formaldehyde, diisocyanate, and 1-ethyl-3- (3-dimethylaminopropyl) carbodiimide. Genipin cross-links materials containing primary amino groups. It can be assumed that genipin crosslinks collagen at the primary amino groups of lysine and 5-hydroxylysine residues [45], however with a lower efficacy than chitosan. On the other hand, admixing chitosan to collagen can form a stable complex and chitosan can act as a binding bridge to increase the efficiency of cross-linking collagen and chitosan due to the sufficient number of primary amino groups in the chitosan molecule [46]. Initially, solutions of 3%, 0.3%, and 0.03% genipin were used to crosslink the gels. Genipin, when used at a concentration of 3%, imparted excessive rigidity to gels, leading to gel fragility and was toxic to cells (data not shown). Therefore, we further used the 0.03% solution of genipin, which crosslinked the gels and restricted the rapid destruction of gels in the solutions.

### 3.4. Cytotoxicity of Gels

**Figure 1:**
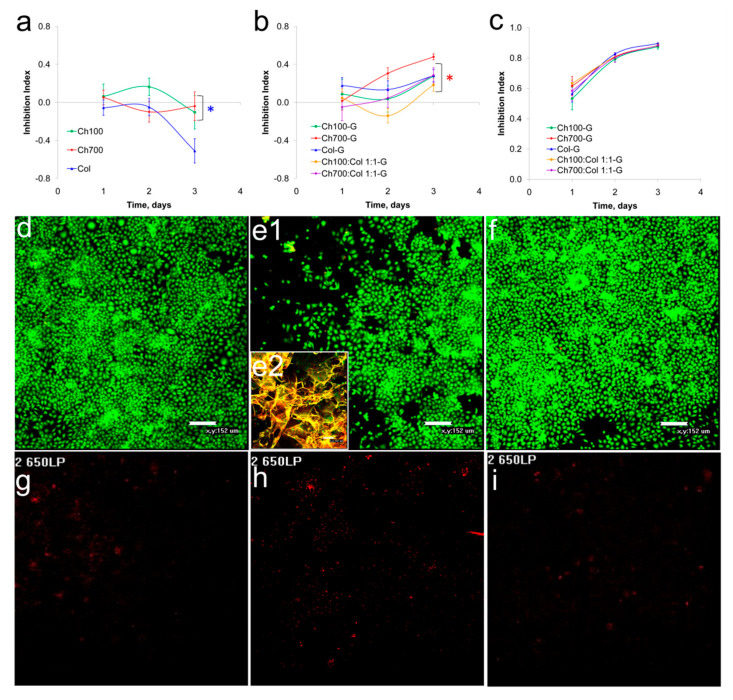
Gels cytotoxicity. (**a**–**c**): Cytotoxicity of uncrosslinked gels (**a**) and gels crosslinked with 0.03% (**b**) and a 0.3% solution of genipin (**c**) on EAhy 926 cell lines. (**d**–**i**) Cells were incubated for 72 h without (**d**,**g**) or with Ch700-G (**e**,**f**,**h**,**i**) and stained with acridine orange (**d**–**f**, green) and propidium iodide (**g**–**i**, red). Insert (**e2**) shows the place of Ch700-G gel deposition in the well. The statistical significance was evaluated using the Student’s *t*-test: Blue star corresponds to—*p* < 0.05 vs. Col, red star—to *p* < 0.05 vs. Ch700-G.

To assess the cytotoxicity of the gels, the MTT test on human endothelial cells EAhy 926 was used. The cytotoxicity of the gels obtained by cross-linking with 0.3% and 0.03% genipin solutions were studied. All gels cross-linked with 0.3% genipin were toxic, gels cross-linked with the 0.03% solution were mostly non-toxic and moderately toxic, in addition, the cytotoxicity of the gels increased over time for cross-linked samples (Figure 1a–c). The data obtained correlate with the results of Sundararaghavan et al., where minimal toxicity was observed at a concentration up to 0.02% of genipin while 0.1% of genipin induced cell death [47]. The cells in the presence of Ch700-G grew almost as in the control wells, except for the area under the gel (Figure 1e1,e2). This area remained cell free, due to a mechanical hindrance and a limited access of CO2 to the cells (Figure 1d–i). No significant increase in apoptotic cell numbers in the cultures with the gels was found (Figure 1g–i). The same data were obtained for the other gels (data not shown).

### 3.5. Swelling Properties of Gels

**Figure 2:**
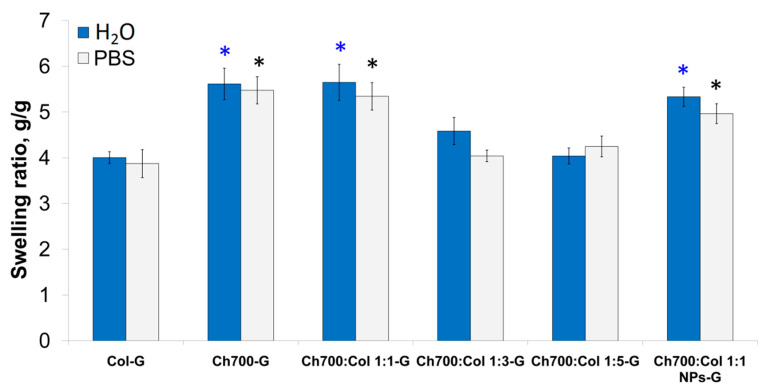
Swelling of gels cross-linked with 0.03% genipin in water or in PBS after 24 h of incubation. The statistical significance was evaluated using the Student’s *t*-test: Blue stars correspond to—*p* < 0.05 vs. Col-G in water, black stars to—*p* < 0.05 vs. Col-G in PBS.

The material for biomedicine must have good swelling ability to absorb exudate. The ability to swell is directly related to the internal structure of the material and is its important property, as it affects the interaction with cells. Collagen and chitosan-based gels and gels containing NPs were swollen in water or PBS. Swelling measurements were performed after 24 h (Figure 2). 

The gels containing more than 50% of Ch700 (Ch700-G, Ch700: Col 1: 1-G, Ch700: Col 1: 1 NPs-G) had better swelling properties compared to collagen-based ones. The high content of Ch700 resulted in a statistically significant increase in swelling capacity (Figure 2). As expected, collagen-based gels had the lowest swelling coefficients. This is possibly due to the absence of ions in this liquid, which can increase the crosslink density of the sample and hinder the swelling process. It should be noted that in the study of swelling, uncrosslinked gels were also used, which quickly disintegrated during the experiment. The poor mechanical properties of collagen also led to the destruction of the porous structure when it was removed from water and the PBS solution. Chitosan with a high molecular weight (Ch700) had greater elasticity, which contributed to the preservation of the original porous structure; with a decrease in molecular weight (100 kDa), the gels gradually dissolved and their weight could not be determined. Therefore, chitosan with a molecular weight of 700 kDa was used for further studies.

### 3.6. Fourier Transform Infrared Spectroscopy (FTIR)

**Figure 3:**
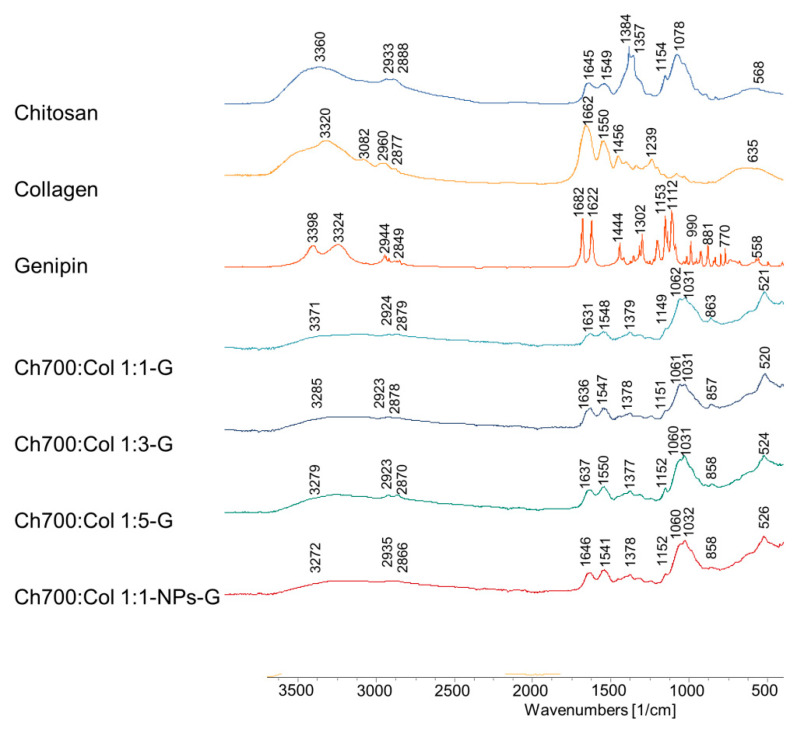
FTIR spectra of Chi, Col, G, Ch700:Col 1:1-G, Ch700:Col 1:3-G, Ch700:Col 1:5-G and Ch700:Col 1:1-NPs-G.

FTIR spectra of gels based on collagen and chitosan cross-linked by genipin are shown in Figure 3. The IR spectra of gels with different ratios of collagen and chitosan practically did not differ from each other. At the same time, characteristic peaks were observed. The secondary amide band observed at 1547 cm^−1^ is an N–H bond and is believed to be due to the formation of secondary amides as a result of the reaction between the ester of the genipin (carboxymethyl groups of the genipin) and the hydroxyl groups and amino groups of chitosan. There are also bands at 1104 and 1370 cm^−1^, which are believed to be vibrations associated with the formation of new bonds between genipin and the primary amines of lysine, hydroxylysine, or arginine residues in collagen. In general, there are no significant differences in the arrangement of bands in the IR spectra of gels with different ratios of chitosan and collagen crosslinked by genipin. Only some changes in the intensity of the bands can be observed.

### 3.7. Scanning Electron Microscopy

**Figure 4:**
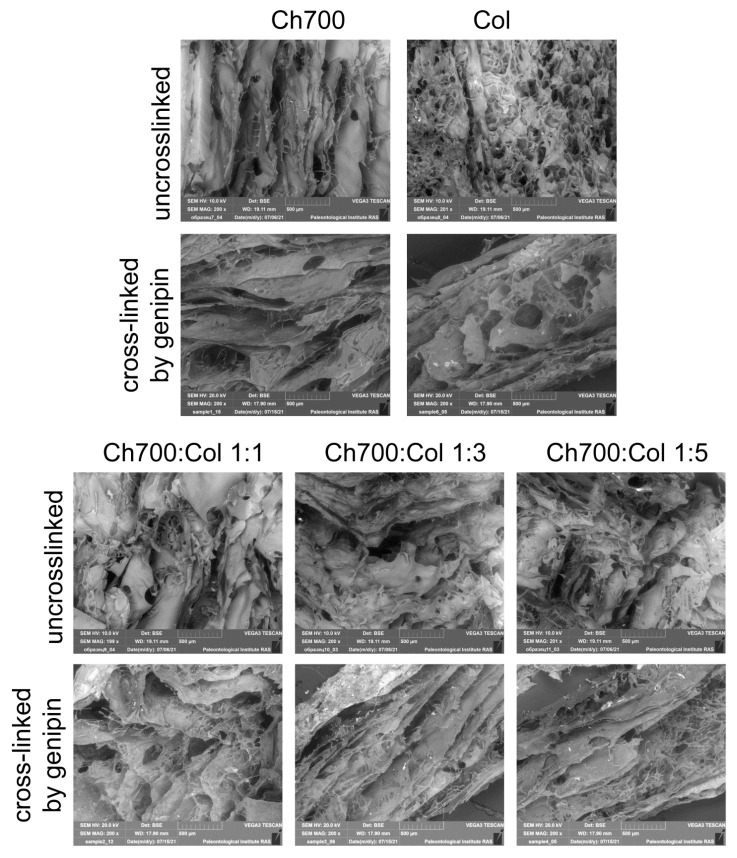
SEM image of the microstructure of the hydrogels with different ratios of collagen and chitosan (Bar = 500 μm).

SEM—cross-sectional images of hydrogels of chitosan, collagen, and gels with different ratios of components uncrosslinked and crosslinked by genipin are shown in Figure 4. 

SEM was used to monitor the morphology of the hydrogels. It has been shown that hydrogels have a fairly uniform macroporous structure. The three-dimensional spatial structure promotes free diffusion of water molecules, and its solid-frame structure allows the hydrogel to absorb a lot of water without destruction. Since chitosan has a higher mechanical strength than collagen, chitosan gels can retain their original structure when crosslinked with genipin [46].

Chitosan gels had a fibrous, banded structure and large pores before and after crosslinking. During the crosslinking procedure, the gels containing chitosan had many crosslinking points, so the small sheets joined together to form larger sheets and cause larger pores to form after repeated lyophilization.

The collagen-based gel, in contrast to chitosan gels, was characterized by small and frequent pores before cross-linking, and after cross-linking with genipin, the structure of the gel also became fibrous and streaky. This change in the structure of the collagen-based gel occurred with the simultaneous crosslinking and rehydration by immersion in the genipin solution. Rehydration caused the porous structure to break down and the fibers to fusion before the genipin began to crosslink. Therefore, the collagen gel formed a typical membrane-like structure [46]. Large pores of the hydrogel observed in all cross-linked samples are favorable for cell proliferation and interaction with them [48].

### 3.8. Rheological Measurements

**Figure 5:**
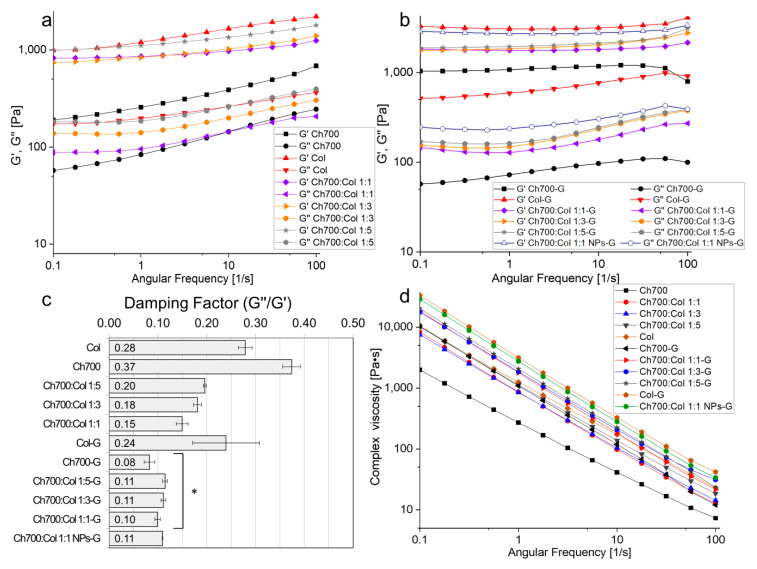
Viscoelastic behavior of different hydrogels. (**a**) Dependence of G’ and G” moduli as functions of angular frequency for gels without genipin. (**b**) Dependence of G’ and G” moduli as functions of angular frequency for genipin-added gels. (**c**) Damping factor at an angular frequency of 10 rad/s, *—*p* < 0.05 vs. uncross-linked gels. (**d**) Complex viscosity of hydrogels as a function of angular frequency.

The study of the viscoelastic properties of materials makes it possible to additionally assess the change in their structure [49,50]. The rheological behavior of gels based on chitosan (MW 700 kDa) and collagen was studied, and the results are shown in Figure 5. The viscoelastic parameters of the swollen gels were investigated: The values of the storage and loss moduli and the complex viscosity were determined as a function of angular frequency. It is known [51,52] that G’ is the storage (elastic) modulus, which indicates the accumulated strain energy that can be returned. G’’ is the loss (viscosity) modulus, which is the deformation energy dissipated during internal friction during flow. If the substance is viscous: G’ = 0 (G” = G*), and if the substance is absolutely elastic: G” = 0, (G’ = G*). G* is the complex modulus, which represents the total sample’s resistance to the applied deformation (G* = G’+ iG”).

A more than two-fold increase in the storage modulus G’ was observed for all samples after the addition of genipin to the gel. This indicates an improvement in elastic properties in the process of cross-linking with genipin. Figure 5a,b show that the storage moduli G’ for all samples are higher than the loss moduli G”, which means that all samples are viscoelastic bodies, not liquids. It should be noted that the curves of storage and loss moduli do not intersect for all samples, which means that the sol-gel transition did not occur over the entire frequency range from 0.1 to 100 rad/s [53].

It was found that with an increase in the amount of collagen in the gels, their storage modules also increased. Thus, for samples without genipin (Figure 5a) at an angular frequency of 10 rad/s, an increase in G’ values was found in the following order: Ch700 < Ch700:Col 1:1 < Ch700:Col 1:3 < Ch700:Col 1:5 < Col. This dependence was also observed for loss moduli.

For genipin crosslinked gels, G’ was changed similarly (Figure 5b). However, the curves G’ and G” had a smaller slope, i.e., there were no significant changes in the values of the moduli when changing the angular velocity. This confirms the efficiency of the genipin crosslinking process because when the angular frequency was changed from 0.1 to 100 Hz in the frequency test, the gel structure was not disturbed. Consequently, the gels remained viscoelastic and did not turn into viscous liquids [54].

The addition of silver nanoparticles to the gels composition increased the storage and loss moduli of the sample crosslinked by genipin Ch700:Col 1:1-G by 1.5 times. The introduction of NPs into gels makes them harder. This is consistent with the data shown in work [55], which reports that the G’ in the viscoelastic region for the hydrogel containing NPs was also greater than that measured for the control gel without nanoparticles. This can be interpreted as an increase in the ability of the Ch700:Col 1:1 NPs-G gel to accumulate deformation energy elastically, which directly correlates with the crosslinking degree within the gel, which leads to a higher mechanical strength [52]. These results indicate that NPs are involved in the formation of a cross-linked polymer matrix, and that the increase in elastic modulus compared to a control gel of the same composition (without nanoparticles) is due to the presence of additional cross-linking provided by nanoparticles.

Complex viscosity is the ratio of the complex modulus to the angular velocity and it reflects the total resistance to dynamic shear. From the results presented in Figure 5d, it can be seen that the viscosity decreases significantly with an increasing angular frequency (about 3 orders of magnitude). A decrease in viscosity values indicates an increase in the gel’s fluidity with an increase in the shear rate. 

Assuming that all viscoelastic gels were reasonably stable at 10 rad/s and showed no intersection of G’ and G”, a frequency of 10 rad/s was used to determine the damping factor (G”/G’). All gel samples can be classified as “weak”, because the damping factor at a selected angular frequency (Figure 5c) is close to 0.1. In [56], it is reported that a low value of the ratio of the loss modulus to the storage modulus makes it possible to distinguish a strong gel (0.01 and below) from a weak (about 0.1). For samples cross-linked with genipin, the value of the G”/G’ is 2–4 times lower than for non-cross-linked ones. This means that such gels are stronger, that is, more elastic. An exception is collagen gel, for which the G”/G’ values are slightly higher than those for uncrosslinked collagen gel (0.239 and 0.279, respectively). Presumably, this can be explained by the fact that genipin in gels interacts mainly with amino groups in chitosan. This is also indirectly confirmed by the intensity of the color of the gel; a less saturated color in gels consist only of collagen. The fact that crosslinked gels are stronger is also evidenced by the practically unchanged values of the storage and loss moduli with a change in frequency, as was shown earlier.

### 3.9. Wound Healing

Wound healing is a multistep process involving a number of interdependent stages such as hemostasis, inflammation, proliferation, and remodeling. Each stage is characterized by its own physiological and biochemical parameters, in which various types of cells and growth factors are involved [57]. This process can be faster when stimulated with materials that accelerate wound healing. However, as a rule, one material does not have all the required properties. Therefore, the gel used in this work was multicomponent; we used collagen, chitosan, and silver nanoparticles in its composition. Chitosan can accelerate wound healing by decreasing the time of inflammation via stimulating different inflammatory cells [11,58,59]. Collagen, when the skin is damaged, causes the activation and aggregation of platelets, forming a fibrin clot. At the inflammatory stage, activation of immune cells stimulates the secretion of pro-inflammatory cytokines, which affect the migration of fibroblasts, epithelial, and endothelial cells. Fibroblasts promote collagen deposition. At the same time, collagen degradation releases fragments that promote fibroblast proliferation and the synthesis of growth factors, which lead to angiogenesis and re-epithelialization [60]. AgNPs were introduced into the gel to increase the antibacterial, antioxidant, and anti-inflammatory activity of the gel [61,62].

**Figure 6:**
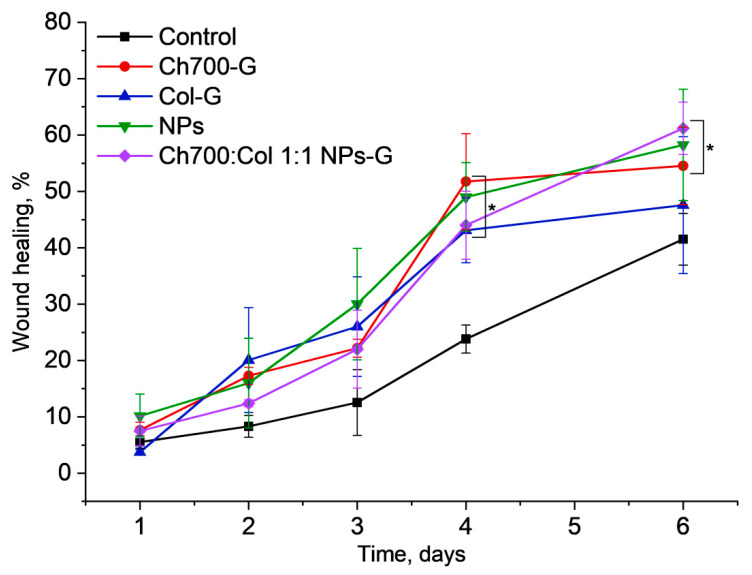
Wound closure without (black curve) or with the gels based on chitosan (Ch700-G), collagen (Col-G), NPs, and Chi700:Col 1:1 NPs-G. The statistical significance was evaluated using the Mann–Whitney test: * *p* < 0.05 vs. control mice.

The wound healing effect was studied in male CD1 mice with induced diabetes. The wounds were bilateral: One with the gel and the other served as a control. Mice, were observed daily and the wounds were measured with a caliper on the 1st, 4th, and 6th days of treatment. A significant effect in the level of wound healing was observed as a rule on the 4th day in all groups of mice. This is in agreement with earlier shown data [63]. Mice treated with gels cross-linked with genipin based on chitosan, chitosan with collagen in a 1:1 ratio with and without NPs, as well as a solution of NPs, showed better wound healing rates starting from day 4 and were more favorable for healing wounds (Figure 6).

### 3.10. Genes Expression

Tissue samples taken near the wound were used to study gene expression. Based on the published data, we have selected the genes of proinflammatory cytokine IL-1b, activators of angiogenesis VEGF, TGF-b1, and MMP-1, MMP-9, MMP-12, and TIMP-1 which are responsible for fibrin matrix cleavage [64,65,66,67,68]. Among the selected genes expression MMP-1 and MMP-9 were not detected (data not shown).

**Figure 7:**
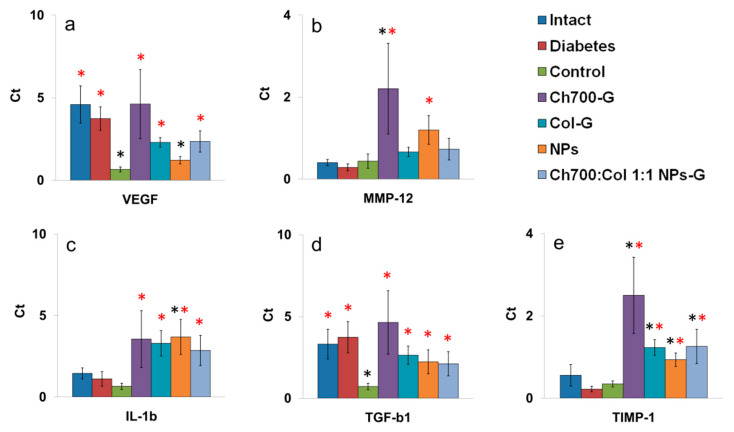
Expression levels of VEGF (**a**), MMP-12 (**b**), IL-1b (**c**), TGF-b1 (**d**), and TIMP-1 (**e**) in skin biopsies of intact mice, diabetic mice without wounds (diabetes), diabetic mice with wounds (control), and diabetic mice with wounds treated by different materials (Ch700-G, Col-G, NPs, Ch700:Col 1:1 NPs-G). The statistical significance was evaluated using the Mann–Whitney test: Black stars—*p* < 0.05 vs. intact mice; red stars—*p* < 0.05 vs. diabetic mice with wounds (control).

The induction of diabetes did not affect the expression level of the studied genes in a comparison with intact mice (Figure 7a–d). Expression of VEGF and TGF-b1 in control wounds decreased in a comparison with intact or diabetic skin which is in agreement with [64,65] where it was shown that the levels of VEGF and TGF-b1 in the damaged skin of diabetic mice were significantly reduced.

After the application of wound healing, materials other than NPs expression of VEGF and TGF-b1 in wound tissue biopsies restored to a value corresponding to intact mice, which may contribute to accelerated wound healing compared to diabetic mice. It is well known that TGF-b1 plays an important role in wound healing. It induces collagen synthesis, angiogenesis, facilitates re-epithelialization, greatly increasing the migration of keratinocytes into the damaged area [66]. VEGF is recognized as an important mediator of angiogenesis. Like TGF-b1, VEGF promotes collagen deposition and epithelialization during wound healing [67]. 

The use of all test materials as wound healing agents led to a significant increase in the expression of IL-1b (Figure 7c). The role of IL-1b in wound healing is that it can directly stimulate collagen secretion by fibroblasts in a dose-dependent manner [68], thereby accelerating the wound healing process.

Matrix metalloproteinases (MMPs) usually are present in both acute and chronic wounds. Endogenous regulators of MMPs activity are TIMPs. TIMP-1 inhibits most types of MMPs, and is present in epithelial cells of excised and burn tissue of wounds [69]. MMPs and TIMPs play a key role in regulating the degradation and deposition of the extracellular matrix, which is important for wound re-epithelialization. Excessive activity of MMPs can lead to chronic non-healing wounds [69]. MMPs mediate the destruction of damaged tissue early in the healing process while at the later stages, MMPs inhibit the formation of new tissue. At these stages, TIMPs play an important role by suppressing the activity of MMPs [70].

Among all MMPs, MMP-12 is macrophage metalloelastase able to degrade elastin, type IV collagen, fibronectin, laminin-1, and other matrix proteins. The level of MMP-12 gene expression corresponds to macrophage migration into the wound tissue. Among all gels, only Ch700-G and NPs significantly stimulated the MMP-12 expression (Figure 7b). Multicomponent gel, which contains both Ch700-G and NPs has lost stimulating activity, possibly due to the collagen effect. Expression TIMP-1 was significantly increased in the treated wounds (Figure 7e).

The most significant increase in gene expression was induced by Ch700-G gel (Figure 7). We hypothesized that this effect was connected with its higher toxicity at high concentrations (Figure 1b). All other gels demonstrated a comparable effect on gene expression.

### 3.11. Histological Analysis of Wound Tissue

**Figure 8:**
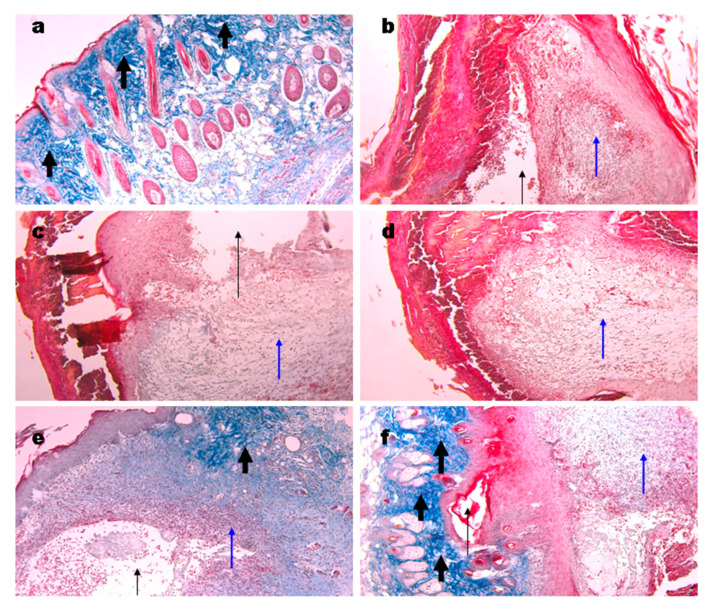
Representative images of the Masson’s trichrome staining of the wound biopsies. Histology of normal skin (**a**), at days 4 (**b**), 5 (**c**), 6 (**d**), 7 (**e**), and 10 (**f**) of wound controls. Thin black arrows show open cavities; thin blue arrows show granulomatous tissue; thick black arrows show collagen deposition. Magnification 100×.

Biopsies of the wounds in the control groups were taken at different days post-surgery. From day 4 to 6, granulomatos tissue of a bright red color and coagulated blood were observed (Figure 8, blue arrows). Open wound cavities were present (Figure 8b–d, black thin arrows) which were slowly filled with the granulomatos tissue. No signs of collagen were found. At day 7, collagen deposition appeared (Figure 8e, thick arrows). At day 10, a small cavity was still present, excessive granulomatos tissue was still observed under the skin which, on the whole, has normal morphology (Figure 8f).

**Figure 9:**
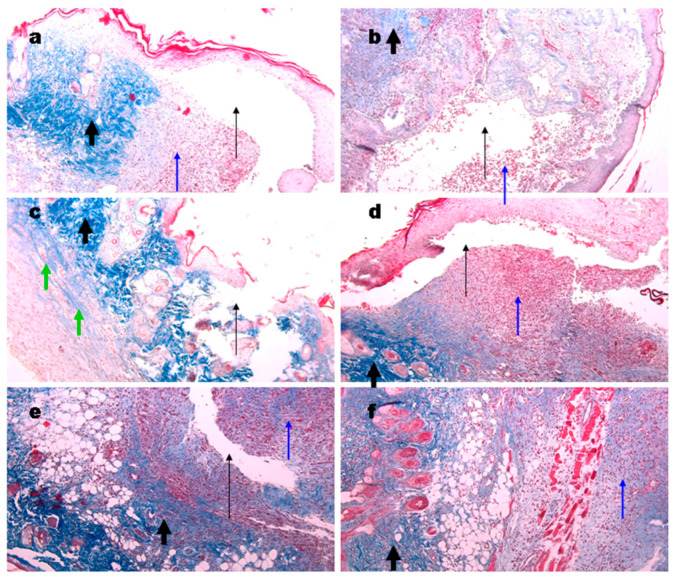
Representative images of the Masson’s trichrome staining of the wound biopsies in mice treated with gels. Wounds at day 6 using Ch700-G (**a**), Ch700-Col 1:1 (**b**), Col (**c**), and NPs (**d**). Day 10 wounds in control (**e**) and in Col mice (**f**). Thin black arrows show open cavities; thick black arrows show collagen deposition; thin blue arrows show granulomatous tissue; and green arrows show collagen originated from the gels. Magnification 100×.

Wound biopsies from mice treated with the gels were obtained at days 6 and 10. We found cavities in all groups treated (Figure 9a–d) while they had a superficial location not like deep cavities in the control group (Figure 8). These cavities are likely to appear during slide preparation due to a lower adhesion of the gels to wound tissue. In all samples, collagen deposition was evident (Figure 9, thick black arrows). No significant differences between the gels used were found. The structure of gel originated collagen was different from the native murine one (green arrows) and it was better seen in the biopsy of the wound treated with collagen gel (Figure 9c). At day 10, the control wound still had small cavities while the gel treated ones were denser however still contained sufficient quantity of granulomatous tissue (Figure 9f, blue arrow). Gel application stimulated hair follicle repair and sebaceous glands formation, which corresponds to the earlier published data [71,72].

## 4. Conclusions

We developed hydrogels based on chitosan and collagen cross-linked by genipin as well as silver nanoparticles coated by chitosan derivative. The gels formed by 700 kDa chitosan and collagen swelled better than gels based on collagen and 100 kDa chitosan only. A fibrous, streaky structure and large pores were observed after the crosslinking of all gels, which is a favorable factor for the interaction with the cells. For hydrogel samples before and after the addition of genipin, an increase in the storage modulus G’ was observed for all gel samples by more than 2 times after crosslinking, which indicates an improvement in elastic properties during genipin crosslinking. The addition of silver nanoparticles to the composition of the gels increased the storage and loss moduli of the sample Ch700:Col 1:1-G by 1.5 times. In the study of wound healing, it was determined that mice treated with gels cross-linked with genipin with and without NPs as well as NPs alone showed a comparable healing effect in a comparison with untreated wounds. All gels increased collagen deposition, hair follicle repair, and sebaceous glands formation. Gels application increased VEGF, TGF-b1, IL-1b, and TIMP1 gene expression, all of which take part in tissue regeneration. The absence of the difference in wound closure between the gels can be explained by the limited ability of the wounded tissue to repair. Animal models do not reflect a complicated process of diabetic ulcers repair in humans. It is likely that different gels will be effective to treat diabetic ulcers depending on their severity. 

## Figures and Tables

**Table 1:** Primers used in the work.

N	Gene	Forward Primer	Reverse Primer
1	GAPDH	TGCACCACCAACTGCTTAG	GGATGCAGGGATGATGTTC
2	VEGF	AGGATGTCCTCACTCGGATG	CTGTCCTCTTGACTCAGGGC
3	TGF-b1	ACTGGAGTTGTACGGCAGTG	GGGGCTGATCCCGTTGATTT
4	IL-1b	TGCCACCTTTTGACAGTGATG	GGAGCCTGTAGTGCAGTTGT
5	MMP-1	GTGAATGGCAAGGAGATGATGG	ACGAGGATTGTTGTGAGTAATGG
6	MMP-9	GGGTCTAGGCCCAGAGGTAA	AGACACGCCCCTTGCTGA
5	MMP-12	TGCACTCTGCTGAAAGGAGTC	TGAGTTGTCCAGTTGCCCAG
6	TIMP-1	GGACCTGGTCATAAGGGCTA	GGCATATCCACAGAGGCTTT

GAPDH—Glyceraldehyde 3-phosphate dehydrogenase. VEGF—Vascular endothelial growth factor. TGF-b1—Transforming growth factor b1. IL-1b—Interleukin 1b. MMP-1—Matrix metalloproteinase 1. MMP-9—Matrix metalloproteinase 9. MMP-12—Matrix metalloproteinase 12. TIMP-1—Tissue metalloproteinase inhibitor 1.

**Table 2:** Influence of the concentration of nitric acid during hydrolysis of chitosan * on the characteristics of the obtained samples.

	HNO_3_, %	Mw, kDa	Ip	DD,%
1	6.5	28	2.00	93
2	3.25	54	2.25	93
3	1.95	100	2.22	93
4	0.65	700	1.20	92

* 5 g of chitosan, 100 mL of HNO_3._

**Table 3:** Minimal inhibitory concentration of chitosans, μg/mL.

	Strain	*S. epidermidis*	*S. aureus*	*E. coli*	*C. albicans*
Sample					
100 kDa		125	>500	≥500	125
700 kDa		250	>500	500	31.25

**Table 4:** List of abbreviations for the hydrogels obtained using different ratios of chitosan and collagen (1:1, 1:3, 1:5) crosslinked with a 0.03% genipin solution (G).

Nomenclature	Gel Composition
Col	Collagen, 1% solution in acetic acid
Ch100	Chitosan (100 kDa), 1% solution in acetic acid
Ch700	Chitosan (700 kDa), 1% solution in acetic acid
Ch100:Col 1:1; Ch100:Col 1:3; Ch100:Col 1:5	Chitosan (100 kDa) and collagen in ratios 1:1, 1:3, 1:5 (mass)
Ch700:Col 1:1; Ch700:Col 1:3; Ch700:Col 1:5	Chitosan (700 kDa) and collagen in ratios 1:1, 1:3, 1:5 (mass)
Col-G	Collagen crosslinked with genipin
Ch100-G	Chitosan (100 kDa) crosslinked with genipin
Ch700-G	Chitosan (700 kDa) crosslinked with genipin
Ch100:Col 1:1-G; Ch100:Col 1:3-G; Ch100:Col 1:5-G	Chitosan (100 kDa) and collagen in ratios 1:1, 1:3, 1:5 (mass) crosslinked with genipin
Ch700:Col 1:1-G; Ch700:Col 1:3-G; Ch700:Col 1:5-G	Chitosan (700 kDa) and collagen in ratios 1:1, 1:3, 1:5 (mass) crosslinked with genipin
Ch700:Col 1:1NPs-G	Chitosan (700 kDa) and collagen in 1:1 ratios with silver nanoparticles synthesized in a medium of quaternized chitosan with gallic acid, crosslinked with genipin

## Data Availability

Data are contained within the article and Supplementary Materials.

## Supplementary Materials

The following are available online at https://www.mdpi.com/article/10.3390/ma15010015/s1, Figure S1: TEM images of AgNPs, Table S1: Average data for particle size distribution of AgNPs, Figure S2: Diameter of the size distribution of AgNPs.
