# Correction: Shagdarova et al. Collagen/Chitosan Gels Cross-Linked with Genipin for Wound Healing in Mice with Induced Diabetes. *Materials* 2022, *15*, 15

**DOI:** 10.3390/ma18235285

**Published:** 2025-11-24

**Authors:** Balzhima Shagdarova, Mariya Konovalova, Yuliya Zhuikova, Alexey Lunkov, Vsevolod Zhuikov, Dolgor Khaydapova, Alla Il’ina, Elena Svirshchevskaya, Valery Varlamov

**Affiliations:** 1 Research Center of Biotechnology, Russian Academy of Sciences, 119071 Moscow, Russia; shagdarova.bal@gmail.com (B.S.); zhuikova.uv@gmail.com (Y.Z.); fwnf1994@gmail.com (A.L.); vsevolod1905@yandex.ru (V.Z.); ilyina@biengi.ac.ru (A.I.); 2 Shemyakin-Ovchinnikov Institute of Bioorganic Chemistry, Russian Academy of Sciences, 117997 Moscow, Russia; mariya.v.konovalova@gmail.com (M.K.); esvir@ibch.ru (E.S.); 3 Faculty of Soil Science, M.V. Lomonosov Moscow State University, 119234 Moscow, Russia; dkhaydapova@yandex.ru

In the original publication [1], there was an overlap in Figure 9 as published. The corrected Figure 9 appears below. The authors state that the scientific conclusions are unaffected. This correction was approved by the Academic Editor. The original publication has also been updated.

## Figures and Tables

**Figure 9:**
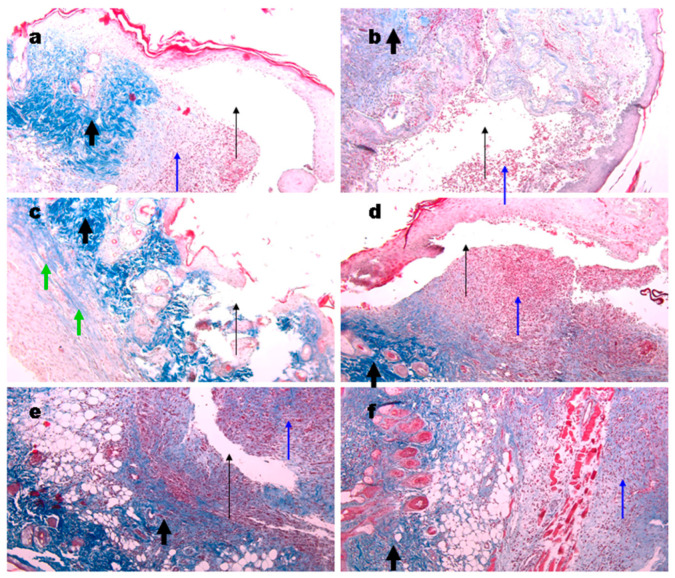
Representative images of the Masson’s trichrome staining of the wound biopsies in mice treated with gels. Wounds at day 6 using Ch700-G (**a**), Ch700-Col 1:1 (**b**), Col (**c**), and NPs (**d**). Day 10 wounds in control (**e**) and in Col mice (**f**). Thin black arrows show open cavities; thick black arrows show collagen deposition; thin blue arrows show granulomatous tissue; and green arrows show collagen originated from the gels. Magnification 100×.

## References

[1] Shagdarova B., Konovalova M., Zhuikova Y., Lunkov A., Zhuikov V., Khaydapova D., Il’ina A., Svirshchevskaya E., Varlamov V. (2022). Collagen/Chitosan Gels Cross-Linked with Genipin for Wound Healing in Mice with Induced Diabetes. Materials.

