# Supplementary material for: Collagen/Chitosan Gels Cross-Linked with Genipin for Wound Healing in Mice with Induced Diabetes
Source: Materials (Basel). 2021 Dec 22;15(1):15. doi: 10.3390/ma15010015 (PMC8745956; doi:10.3390/ma15010015)
Supplement: Supplementary file 1 [file materials-15-00015-s001.zip › materials-1501974-supplementary.pdf]

materials

MDPI

Article

Collagen/chitosan gels cross-linked with genipin for wound healing in mice with induced diabetes

Balzhima Shagdarova[1], Mariya Konovalova[2], Yuliya Zhuikova[1], Alexey Lunkov[1], Vsevolod Zhuikov[1], Dolgor Khaydapova[3], Alla Il'ina[1], Elena Svirshchevskaya[2] and Valery Varlamov[1,*]

Research Center of Biotechnology, Russian Academy of Sciences, 119071 Moscow, Russia; shagdarova.bal@gmail.com (B.S.); zhuikova.uv@gmail.com (Y.Z.); fwnf1994@gmail.com (A.L.); vsevolod1905@yandex.ru (V.Z.); ilyina@biengi.ac.ru (A.I.)
[2] Shemyakin-Ovchinnikov Institute of Bioorganic Chemistry, Russian Academy of Sciences, 117997 Moscow, Russia; mariya.v.konovalova@gmail.com (M.K.); esvir@ibch.ru (E.S.)
[3] Faculty of Soil Science, M.V. Lomonosov Moscow State University, 119234 Moscow, Russia; dkhaydapova@yandex.ru (D.K.)
* Correspondence: varlamov@biengi.ac.ru

Characterization of silver nanoparticles by TEM and DLS

1. TEM images of AgNPs samples were determined by JEM 1400 (JEOL, Japan) electron microscope, equipped with INCA Energy TEM 350 (Oxford Instruments, UK).The obtained TEM images of nanoparticles were processed to determine dimensional characteristics using ImageJ software (National Institutes of Health, USA). The presence of silver in nanoparticles samples was confirmed by EDX spectra and EDS with elemental mapping.

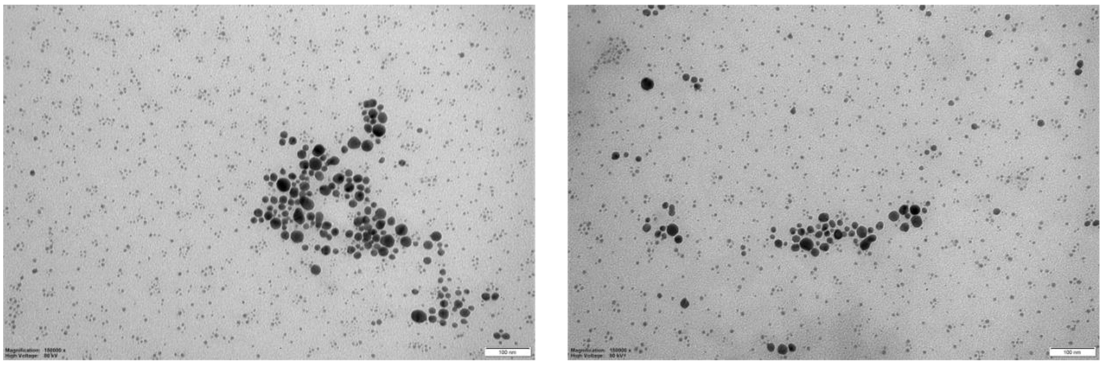

Figure S1. TEM images of AgNPs

2. The average hydrodynamic particle diameter and particle size distribution (polydispersity index, PDI) were determined by dynamic light scattering (DLS) in reflected light (scattering angle 180 °C) using a NANO-flex II analyzer (Colloid Metrix, Germany). The measurements were carried out in distilled water, the sample temperature is + 22 °C, the sample volume is not less than 1 mL. The measurements were carried out in triplicate,

the result was averaged.

Table S1. Average data for particle size distribution of AgNPs

Sample	Mean_I /nm	Z-value /nm	PDI	Mean_N /nm	Peak_N /nm	Peak_I /nm
AgNPs	44.84±0.57	45.92±0.53	0.0762±0.01	30.02±0.48	28.66±0.50	43.44±0.88

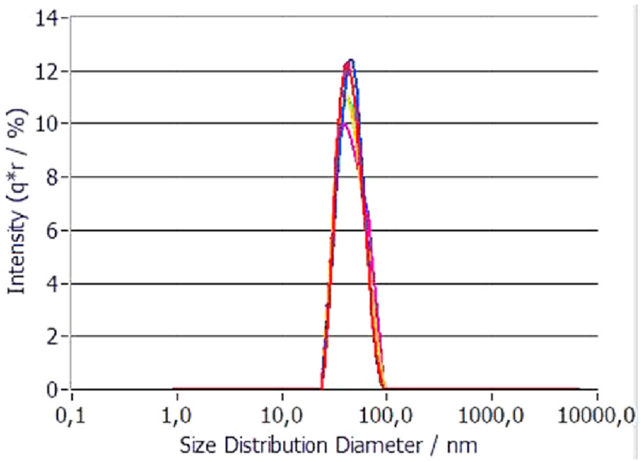

Figure S2. Diameter of the size distribution of AgNPs
